# Human depression: a new approach in quantitative psychiatry

**DOI:** 10.1186/1744-859X-9-25

**Published:** 2010-06-03

**Authors:** Massimo Cocchi, Lucio Tonello, Mark M Rasenick

**Affiliations:** 1 DIMORFIPA, University of Bologna, Italy; 2 Faculty of Veterinary Medicine, University of Bologna, Italy; 3 Faculty of Human Sciences, LUdeS University, Lugano, Switzerland; 4 Department of Physiology and Biophysics, University of Illinois, Chicago College of Medicine, Chicago, IL, USA

## Abstract

The biomolecular approach to major depression disorder is explained by the different steps that involve cell membrane viscosity, Gsα protein and tubulin. For the first time it is hypothesised that a biomolecular pathway exists, moving from cell membrane viscosity through Gsα protein and Tubulin, which can condition the conscious state and is measurable by electroencephalogram study of the brain's γ wave synchrony.

## Introduction

The need for a deep, radical turning point in the world of psychiatry is rapidly growing. Present diagnostic methods cannot continue to be considered acceptable because they are almost completely based on the psychiatrist's opinion, which does not have an objective diagnostic technology and thus has a very high error rate.

A debate is essential between the advocates of traditional diagnostic and therapeutic methods and advocates of emerging methods resulting from new discoveries. Major depressive disorder and other related and non-related psychiatric conditions are still characterised and defined by descriptive and non-biological criteria, but it is hoped that we can adequately characterise this and other psychiatric disorders with the addition of new quantitative approaches.

### Human depression in the interpretation of an artificial neural network

Following the theory that a biomolecular involvement of the cell could be an expression of a psychiatric disorder, we have tried to understand and explain this phenomenon.

The intention was to study the platelet fatty acids composition in normal and depressed subjects [1], because of their similarity to neurons [2-10].

Membrane platelet fatty acids of subjects with a clinical diagnosis of major depression versus apparently normal subjects were assessed. The complexity of membrane dynamics has also suggested study by means of non-linear advanced analytical tools would be appropriate. In particular, it seemed more appropriate to use artificial neural networks: the self-organising map (SOM) Kohonen network [11-13]. This particular algorithm allows viewing of the result graphically, building a two-dimensional map that places the subjects in a continuous, not necessarily dichotomised way.

The values for fatty acids in the two populations were entered into the SOM, mixing normal and pathological subjects and hiding the information relating to their pathological condition. The SOM was then used to map the two populations using three specific fatty acids: palmitic acid (PA), linoleic acid (LA) and arachidonic acid (AA), which represent the majority of total membrane fatty acids, recognising as similar those belonging to the same population and then separating the normal cases from the pathological cases [1]. All the artificial neural networks (ANNs) tested gave essentially the same result. However, the SOM gave superior information by allowing the results to be described in a two-dimensional plane with potentially informative border areas. The central property of the SOM is that it forms a non-linear projection of a high-dimensional data manifold on a regular, low-dimensional (usually 2D) grid.

**Figure 1:**
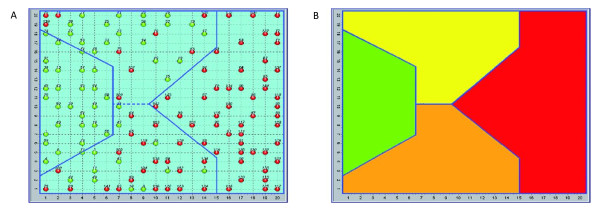
**Distribution of the human subjects (normal and depressive) over the self-organising map (SOM) (a), and SOM areas (b)**. (a) The distribution of the 144 subjects studied, 60 apparently healthy (green) and 84 diagnosed as depressed (red) effected by the SOM allowed us to identify 4 areas: 2 specific ones (exclusively normal and exclusively pathological) and 2 mixed with different concentrations of pathological subjects and apparently normal subjects of the sample. The red subjects in the two intermediate areas (yellow and orange) have been interpreted as having a misleading diagnosis of major depression, as described in the literature [14]. (b) SOM areas. Green = normal, red = depressive, yellow = high density of normal subjects, orange = high density of pathological subjects.

This experiment was performed outside of evidence-based medicine (EBM) rules. The direct task of finding biomarkers according to the EBM rules requires the elimination of selection bias, and in psychiatric illness the leads to the selection of a population that is often clinically unrealistic. The results are shown in Figure 1a, b.

The SOM has shown considerable correlation to the clinical diagnosis of major depression, and indeed, revealed the existence of differences within the same diagnosis. The literature shows that a diagnosis of major depression is very often misleading, and can be changed to a diagnosis of bipolar disorder [14].

Using the following equation (Equation 1), which relates each fatty acid percentage with the melting point and the molecular weight, we obtained a result that led us to understand that platelet membranes had different degrees of viscosity and/or unsaturation (B2 index).

Where *A*_*i *_= percentage of *i*-th fatty acid, *mp *= melting point, *mw *= molecular weight, *mw*_*i *_= molecular weight of *i*-th fatty acid, *mp*_*i *_= melting point of *i*-th fatty acid, and *i*:

• 1 = palmitic acid, C 16:0

• 2 = linoleic acid, C 18:2

• 3 = arachidonic acid, C 20:4

The result clearly showed that the platelet membranes of depressive subjects were characterised by a much higher degree of fatty acid unsaturation than normal subjects.

According to Donati *et al. *[15] rapid changes in membrane lipid composition or in the cytoskeleton could modify neuronal signalling. As this could have implications for a new understanding of some aspects of psychiatric disorders, a private meeting was organised in Bologna (Faculty of Veterinary Medicine) and in Treviso, University, October 2008) with some expert scientists in the field (Kary Mullis and Stuart Hameroff).

Three essential points constituted the crucial elements of the discussion at the meeting: (1) the viscosity of the platelet and neuronal membrane; (2) the protein Gsα; (3) the relationship between tubulin and consciousness.

With regard to the first point, Cocchi and Tonello observed that the platelet membrane was substantially differentiated from a chemical point of view with regard to the indexes of saturation between depressed and normal populations [1].

On the second point, the protein Gsα modifies its structure according to the degree of viscosity of the neuronal membrane, as seen in patients who commit suicide for psychiatric reasons in comparison to deaths from other causes [15].

With regard to the third point, Tubulin, because of its connection to Gsα and its position in the cellular cytoskeleton, determines those changes that have been assessed with quantum computation under conditions of wakefulness in comparison to the condition of anaesthesia [16].

### Biomolecular depression hypothesis

**Figure 2:**
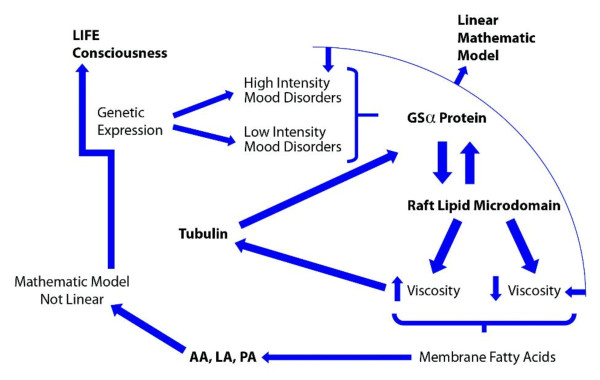
**Description of the biomolecular steps possibly involved in depressive disorder**.

A very suggestive hypothesis was built, as summarised in Figure 2.

**Figure 3:**
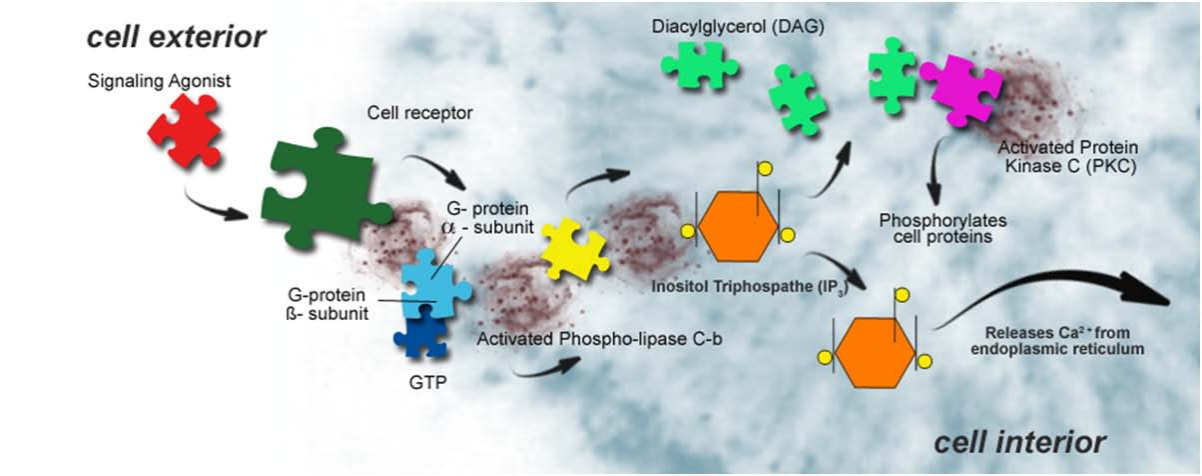
**Description of protein kinase C (PKC) activation**.

Figure 2 describes the molecular depression hypothesis formed according to the experimental findings of Cocchi *et al. *[1], Donati *et al. *[15], Hameroff and Penrose [16,17] and Hameroff [17]. Because of the possible similarity in behaviour of platelets and neurons, membrane viscosity may therefore modify the Gsα protein status. The Gsα protein is connected with tubulin. Tubulin, depending on local membrane lipid phase concentration, may serve as a positive or negative regulator of phosphatidylinositol bisphosphate (PIP2) hydrolysis, as G proteins do. Tubulin is known to form high-affinity complexes with certain G proteins. The formation of such complexes allows tubulin to activate Gα protein, which, in turn, can activate protein kinase C (PKC), and fosters a system whereby elements of the cytoskeleton can influence G-protein signalling. PKC activation (Figure 3) is preceded by a number of steps, originating from the binding of an extracellular ligand that activates G-protein on the cytosolic side of the plasma membrane [18].

**Figure 4:**
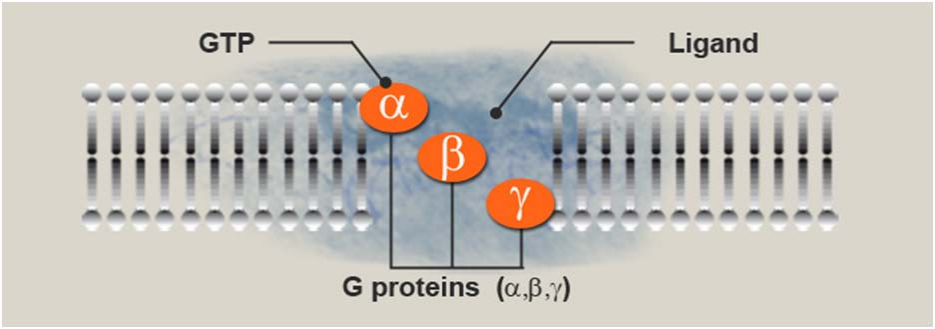
**Ligand reaches the receptor and guanosine-5'-triphosphate (GTP) reaches the protein**. Cell membrane proteins coupled to cell surface receptors bind to GTP upon stimulation of the receptor by an extracellular signalling molecule (as a hormone or neurotransmitter) to form an active complex that mediates an intracellular event (for example, activation of adenylate cyclase).

The G-protein, using guanosine triphosphate (GTP) as an energy source, then activates PKC via the PIP2 intermediate, the diacylglycerol/inositol triphosphate (DAG/IP3) complex [15]. The schematic biomolecular mechanism of the Gsα protein is described in Figure 4.

**Figure 5:**
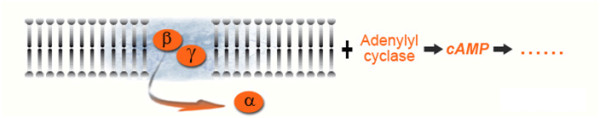
**cAMP signalling pathway**.

The Gα subunit is activated and starts a cAMP signalling cascade, as shown in Figure 5.

The international scientific literature has reported abnormalities in the cAMP signalling cascade of the human brain in suicidal and depressive subjects for over two decades [19-25].

**Figure 6:**
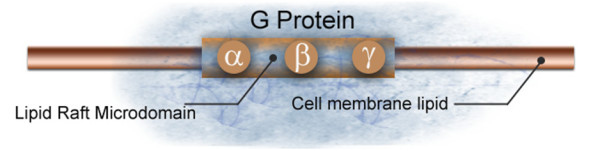
**Schematic representation of a lipid raft microdomain**.

According to Donati *et al. *[15] there is a further possible condition: the position of Gα (Gsα in particular) within the lipid raft microdomain. Lipid rafts are specialised structures on the plasma membrane that have an altered lipid composition as well as links to the cytoskeleton (Figure 6). They are local lipid microdomains that float in the liquid-disordered lipid bilayer of cell membranes. The effect of lipid rafts on neurotransmitter signalling has also been implicated in neurological and psychiatric diseases [26].

Raft localisation of Gsα in human peripheral tissue (possibly platelets, see [15]) may thus serve as a biomarker for depression. Several studies using human platelets suggest that adenylyl cyclase may, in fact, serve as a biological marker for depression [27-34].

### The membrane fatty acid-Gsα hypothesis

It is known that G proteins could be targeted to raft domains by several mechanisms. The most plausible mechanism is that Gα subunits are subject to palmitoylation. Palmitoylation is a process of covalent attachment of palmitic acid to cysteine residues of membrane proteins.

Palmitic acid is one of the three fatty acids (together with arachidonic acid and linoleic acid) used by SOM as marker of depression [1].

**Figure 7:**
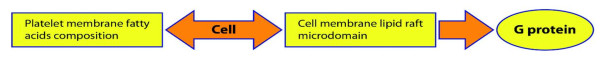
**Schematic description of the possible link between the two research projects on platelet fatty acids and Gsα protein **[1,14].

*Is the critical composition of the membrane platelet fatty acids an indirect measure of the G protein status? (see Figure *7.)

Rapid changes in membrane lipid composition or in the cytoskeleton might modify neuronal signalling. Hameroff hypothesised that through this mechanism it is possible to modify the consciousness state [16,17]. According to Hameroff [16,17] the best measurable correlate of consciousness is a γ synchrony electroencephalogram (γ waves are a pattern of brain waves, with a frequency between 25 to 100 Hz, prototypical at 40 Hz), which indeed rapidly moves and redistributes throughout the brain. γ Synchrony derives not from neurocomputation, but from groups of neuronal dendrites (and glia) transiently fused by electrical synapses called gap junctions, more or less sideways to the flow of neurocomputation. The process could be mediated by tubulin and its correlates i.e. membrane viscosity and Gsα protein (see Figure 2).

Recent studies reported a model of the disconnection hypothesis of schizophrenia through the demonstration of abnormal stimulus induced γ phase synchrony [35].

The idea discussed by the authors with Hameroff and Mullis that platelets could represent the peripheral markers of the depressive disorder and that platelets are 'brain ambassadors', has become a more and more realistic proposal [36].

## Conclusions

On the basis of the above-cited research it is possible to try to understand and quantify some of the biological aspects that characterise depression in order to enable an objective diagnosis to be made through simple and inexpensive blood tests. Such tests, and the biomolecular pathways upon which they are based, would also represent early indicators of therapeutic effectiveness. These possibilities represent a genuine revolution not only in psychiatry but more generally in the worlds of neuroscience and medicine, as Mullis and Hameroff have highlighted in a recent interview on the subject [37,38].

Observed changes in the serotonergic and microtubular systems in the hippocampus following restraint stress confirm the structural [39,40] and biochemical [41] vulnerability of this area to stressful conditions. Cytoskeletal changes represent a potential new pathway that may increase our understanding of psychiatric disorders. The question of whether or not changes in 5-hydroxytryptamine (5-HT)-serotonin levels are related to changes in the expression of tubulin needs to be assessed by future studies [42]. Already in 1980 it has been shown a relationship between serotonin receptors and lipid membrane fluidity: as the membrane lipids become more viscous, the specific binding of serotonin increases steadily. Signal transduction, either through activation of adenylate cyclase by the ligand-receptor complex or by microaggregation of ligand-receptor complexes, is associated with lateral movements of components of the membrane which are determined, at least partially, by lipid fluidity [43]. Since it is well known that Gsα protein and tubulin have a connexion [44] it seemed to us reasonable to raise the question of a possible link to consciousness according to Hameroff-Penrose Orch theory [16,17]. The results will have practical use and be of great interest in more than one scientific field of application e.g, in the study of new drugs for psychiatric disorders and in the diagnostic evaluation of depressive disorders.

## Competing interests

The authors declare that they have no competing interests.

## Authors' contributions

All the authors made substantial contributions to the design and concept of the study. MC and LT were particularly involved in data collection and data analysis. All authors were involved in the interpretation of the data. All the authors have been involved in drafting and revising the manuscript and have read and approved the final manuscript.

## References

[B1] Cocchi M Tonello L Tsaluchidu S Puri BK The use of artificial neural networks to study fatty acids in neuropsychiatric disorders BMC Psychiatry 2008 8 Suppl 1 S3 10.1186/1471-244X-8-S1-S3 18433513 PMC2330078

[B2] Coleman M Platelet serotonin in disturbed monkeys and children Clin Proc Children's Hospital 1971 27 187 194

[B3] Takahashi S Reduction of blood platelet serotonin levels in manic and depressed patients Folia Psychiatr Neurol Jpn 1976 30 475 486 1021543 10.1111/j.1440-1819.1976.tb02670.x

[B4] Sthal SM The human platelet. A diagnostic and research tool for the study of biogenic amines in psychiatric and neurologic disorders Arch Gen Psychiatry 1977 34 509 516 140632 10.1001/archpsyc.1977.01770170019001

[B5] Kim HL Plaisant O Leboyer M Gay C Kamal L Devynck MA Meyer P Reduction of platelet serotonin in major depression (endogenous depression) C R Seances Acad Sci III 1982 295 619 622 6819060

[B6] Dreux C Launay JM Blood platelets: neuronal model in psychiatric disorders Encephale 1985 11 57 64 2862006

[B7] Arora RC Meltzer HY Increased serotonin 2 (5-HT2) receptor binding as measured by 3H-lysergic acid diethylamide (3H-LSD) in the blood platelets of depressed patients Life Sci 1989 44 725 734 10.1016/0024-3205(89)90384-6 2927243

[B8] Thompson P Platelet and erythrocyte membrane and fluidity changes in alcohol dependent patients undergoing acute withdrawal Alcohol Alcoholism 1999 3 349 354 10.1093/alcalc/34.3.349 10414610

[B9] Camacho A Dimsdale JE Platelets and psychiatry: lessons learned from old and new studies Psychosom Med 2000 62 326 336 10845346 10.1097/00006842-200005000-00006

[B10] Plein H Berk M The platelet as a peripheral marker in psychiatric illness Clin Exp Pharmacol 2001 16 229 236 10.1002/hup.251 12404575

[B11] Kohonen T Self-organized formation of topologically correct feature maps Biol Cybern 1982 43 59 69 10.1007/BF00337288

[B12] Kohonen T Self-Organizing Maps 2001 3 Berlin, Germany: Springer

[B13] Kohonen T Kaski S Somervuo P Lagus K Oja M Paatero V Self-organizing map Neurocomputing 1998 21 113 122

[B14] Bowden CL Strategies to reduce misdiagnosis of bipolar depression Psychiatr Serv 2001 52 51 55 10.1176/appi.ps.52.1.51 11141528

[B15] Donati RJ Dwivedi Y Roberts RC Conley RR Pandey GN Rasenick MM Postmortem brain tissue of depressed suicides reveals increased Gs localization in lipid raft domains where it is less likely to activate adenylyl cyclase J Neurosci 2008 28 3042 3050 10.1523/JNEUROSCI.5713-07.2008 18354007 PMC6670711

[B16] Hameroff SR Penrose R Hameroff SR, Kaszniak A, Scott AC Orchestrated reduction of quantum coherence in brain microtubules: a model for consciousness Toward a Science of Consciousness - The First Tucson Discussions and Debates 1996 Cambridge, MA, USA: MIT Press 507 540

[B17] Hameroff SR The "conscious pilot"-dendritic synchrony moves through the brain to mediate consciousness J Biol Phys 2010 36 71 93 10.1007/s10867-009-9148-x 19669425 PMC2791805

[B18] Alberts B Bray D Lewis J Raff M Roberts K Watson J Molecular Biology of the Cell 1994 Garland Science Publishing, New York and Oxford

[B19] Cowburn RF Marcusson JO Eriksson A Wiehager B O'Neill C Adenylyl cyclase activity and G-protein subunit levels in postmortem frontal cortex of suicide victims Brain Res 1994 633 297 304 10.1016/0006-8993(94)91552-0 8137164

[B20] Pacheco MA Stockmeier C Meltzer HY Overholser JC Dilley GE Jope RS Alterations in phosphoinositide signaling and G-protein levels in depressed suicide brain Brain Res 1996 723 37 45 10.1016/0006-8993(96)00207-7 8813380

[B21] Dowlatshahi D MacQueen GM Wang JF Reiach JS Young LT G protein-coupled cyclic AMP signaling in postmortem brain of subjects with mood disorders: effects of diagnosis, suicide, and treatment at the time of death J Neurochem 1999 73 1121 1126 10.1046/j.1471-4159.1999.0731121.x 10461903

[B22] Stewart RJ Chen B Dowlatshahi D MacQueen GM Young LT Abnormalities in the cAMP signaling pathway in post-mortem brain tissue Brain Res Bull 2001 55 625 629 10.1016/S0361-9230(01)00524-X 11576759

[B23] Dwivedi Y Rizavi HS Conley RR Roberts RC Tamminga CA Pandey GN mRNA and protein expression of selective alpha subunits of G proteins are abnormal in prefrontal cortex of suicide victims Neuropsychopharmacology 2002 27 499 517 12377388 10.1016/S0893-133X(02)00335-4

[B24] Dwivedi Y Rizavi HS Shukla PK Lyons J Faludi G Palkovits M Sarosi A Conley RR Roberts RC Tamminga CA Pandey GN Protein kinase A in postmortem brain of depressed suicide victims: altered expression of specific regulatory and catalytic subunits Biol Psychiatry 2004 55 234 243 10.1016/j.biopsych.2003.11.003 14744463

[B25] Pandey GN Dwivedi Y Ren X Rizavi HS Mondal AC Shukla PK Conley RR Brain region specific alterations in the protein and mRNA levels of protein kinase A subunits in the post-mortem brain of teenage suicide victims Neuropsychopharmacology 2005 30 1548 1556 10.1038/sj.npp.1300765 15920506

[B26] Allen JA Halverson-Tamboli RA Rasenick MM Lipid raft microdomains and neurotransmitter signaling Nature Rev Neurosci 2007 8 128 140 10.1038/nrn2059 17195035

[B27] Mooney JJ Schatzberg AF Cole JO Kizuka PP Salomon M Lerbinger J Pappalardo KM Gerson B Schildkraut JJ Rapid antidepressant response to alprazolam in depressed patients with high catecholamine output and heterologous desensitization of platelet adenylate cyclase Biol Psychiatry 1988 23 543 559 10.1016/0006-3223(88)90002-9 2833319

[B28] Garcia-Sevilla JA Padro D Giralt MT Guimon J Areso P Alpha 2-adrenoceptor-mediated inhibition of platelet adenylate cyclase and induction of aggregation in major depression. Effect of long-term cyclic antidepressant drug treatment Arch Gen Psychiatry 1990 47 125 132 1967926 10.1001/archpsyc.1990.01810140025005

[B29] Pandey GN Pandey SC Davis JM Peripheral adrenergic receptors in affective illness and schizophrenia Pharmacol Toxicol 1990 66 13 36 10.1111/j.1600-0773.1990.tb02071.x 2179928

[B30] Pandey GN Pandey SC Janicak PG Marks RC Davis JM Platelet serotonin-2 receptor binding sites in depression and suicide Biol Psychiatry 1990 28 215 222 10.1016/0006-3223(90)90576-N 2378926

[B31] Menninger JA Tabakoff B Forskolin-stimulated platelet adenylyl cyclase activity is lower in persons with major depression Biol Psychiatry 1997 42 30 38 10.1016/S0006-3223(96)00245-4 9193739

[B32] Mooney JJ Samson JA McHale NL Colodzin R Alpert J Koutsos M Schildkraut JJ Signal transduction by platelet adenylate cyclase: alterations in depressed patients may reflect impairment in the coordinated integration of cellular signals (coincidence detection) Biol Psychiatry 1998 43 574 583 10.1016/S0006-3223(97)00327-2 9564442

[B33] Menninger JA Barón AE Conigrave KM Whitfield JB Saunders JB Helander A Eriksson CJ Grant B Hoffman PL Tabakoff B Platelet adenylyl cyclase activity as a trait marker of alcohol dependence Alcohol Clin Exp Res 2000 24 810 821 10.1111/j.1530-0277.2000.tb02060.x 10888069

[B34] Hines LM Tabakoff B Platelet adenylyl cyclase activity: a biological marker for major depression and recent drug use Biol Psychiatry 2005 58 955 962 10.1016/j.biopsych.2005.05.040 16095566

[B35] Flynn G Alexander D Harris A Whitford T Wong W Galletly C Silverstein S Gordon E Williams LM Increased absolute magnitude of gamma synchrony in first-episode psychosis Schizophr Res 2008 105 262 271 10.1016/j.schres.2008.05.029 18603413

[B36] Cocchi M Tonello L Running the hypothesis of a biomolecular approach to psychiatric disorder characterization and fatty acids therapeutical choices Ann Gen Psychiatry 2010 9 Suppl 1 S26 10.1186/1744-859X-9-S1-S26

[B37] Mullis KB Interview by Marco Pivato. Ma la depressione è nel sangue Newspaper: La Stampa, insert: TuttoScienze 2008 5

[B38] Hameroff SR Interview by Marco Pivato. Ma la depressione è nel sangue Newspaper: La Stampa, insert: TuttoScienze 2008 5

[B39] McEwen BS *Stress *and hippocampal plasticity Annu Rev Neurosci 1999 22 105 122 10.1146/annurev.neuro.22.1.105 10202533

[B40] Duman SR Malberg J Nagakawa S D'Sa C Neuronal plasticity and survival in mood disorder Biol Psychiatry 2000 48 732 739 10.1016/S0006-3223(00)00935-5 11063970

[B41] Chaouloff F Serotonin, stress and corticoids J Psychopharmacol 2000 14 139 151 10.1177/026988110001400203 10890308

[B42] Bianchi M Heidbreder C Crespi F Cytoskeletal changes in the hippocampus following restraint stress: Role of serotonin and microtubules Synapse 2003 49 188 194 10.1002/syn.10230 12774303

[B43] Heron DS Shinitzky M Hershkowitz M Samuel D Lipid fluidity markedly modulates the binding of serotonin to mouse brain membranes Proc Natl Acad Sci 1980 77 7463 7467 10.1073/pnas.77.12.7463 6938985 PMC350524

[B44] Popova JS Greene AK Wang J Rasenick MM Phosphatidylinositol 4, 5-bisphosphate modifies tubulin participation in phospholipase Cβ_1 _signaling J Neurosci 2002 22 1668 1678 11880496 10.1523/JNEUROSCI.22-05-01668.2002 PMC6758882

